# Oral Administration of Interleukin-10 and Anti-IL-1 Antibody Ameliorates Experimental Intestinal Inflammation

**DOI:** 10.4021/gr556w

**Published:** 2013-09-09

**Authors:** Diego Cardani, Giuseppina F Dusio, Patrizia Luchini, Michele Sciarabba, Umberto Solimene, Cristiano Rumio

**Affiliations:** a Department of Medical Biotechnology and Translation Medicine, Universita degli Studi di Milano,Via Vanvitelli 32, 20133 Milan, Italy; b Scott and White Healthcare Temple Texas, Via Celoria 10, 20133 Milan, Italy; c Dipartimento di Scienze Veterinarie per la Salute, la Produzione Animale e la Sicurezza Alimentare, Via Celoria 10, 20133 Milan, Italy; d Dipartimento di Informatica e Comunicazione, Universita degli Studi di Milano, Milan, Italy; e Dipartimento di Scienze Biomediche per la Salute, Universita degli Studi di Milano, Via Mangiagalli 31, 20133 Milan, Italy; f WHO Coll. Center for Traditional Medicine, CREBION, Centro Interdipartimentale di Ricerca per lo studio degli Effetti Biologici delle Nano-concentrazioni. Via Celoria 10, 20133 Milan, Italy

**Keywords:** Intestinal bowel disease, Low doses, Interleukin-10, Anti-interleukin-1 alpha antibody, Tight-junctions

## Abstract

**Background:**

To elucidate the effects of a solution containing interleukin-10 and anti-IL-1 antibody in modulating experimental intestinal inflammation.

**Methods:**

Colitis was induced in BALB/c mice by oral administration of dextran sodium sulphate; mice were then treated with interleukin-10 plus anti-IL-1 antibody at low dosage. Transepithelial electrical resistance of isolated mouse colon and colon lengths were evaluated. Cytokines concentrations in organocultures supernatants and plasma samples were evaluated by Enzyme-Linked Immuno Sorbent Assay. Tight junction proteins were evaluated by immunofluorescence, respectively.

**Results:**

Oral administration of tested products restores intestinal barrier function during experimental intestinal inflammation in association with reduced levels of proinflammatory cytokines, increased interleukin-10 plasma concentrations and a tight junction architecture restoration.

**Conclusion:**

Obtained results may contribute to modelling an interesting strategy for the treatment of patients with inflammatory bowel diseases.

## Introduction

Inflammatory bowel diseases (IBDs) are a group of chronic systemic diseases involving inflammation of the gastrointestinal tract. Major forms of IBDs include ulcerative colitis (UC), which affects only the large bowel, and Crohn’s disease (CD), which can affect the entire gastrointestinal tract. The pathogenesis of IBDs is not completely understood, even if current theories hypothesize that IBD-related mucosal inflammation results from an exaggerated reaction of the mucosal immune system against components of the normal intestinal flora [1, 2]. No single infectious microorganism has been identified to cause these diseases, and genetic factors that confer susceptibility to IBDs are being unravelled [3]. Conventional treatments for IBD include corticosteroids, mesalamine, and thiopurines, therapies mostly aimed at blocking downstream inflammatory events. Biological agents, such as antitumor necrosis factor-α (TNF-α) agents Infliximab and Adalimumab, are currently emerging as a highly effective therapy for both UC and CD [4]. The addition of anti-TNF-α antibodies to the therapeutic drugs against Crohn’s disease has been a great innovation in its management. However, approximately 25 to 40% of patients who initially have a good feedback from anti-TNF-α treatment develop severe adverse events or loose their response during maintenance therapy [5-8]. For these reasons, novel agents aimed at dampening IBD-related mucosal inflammation are highly advocated. Pharmacological blockade of proinflammatory mediators and administration of molecules with anti-inflammatory activity (for example, cytokines) are currently considered promising therapeutic approaches for the control of IBD-related mucosal inflammation [9], but it might be necessary to learn how to use them with efficacy and safety: for example, oral administration of cytokines can avoid the deleterious consequences of systemic route, retaining sufficient biological activity to exert immunomodulatory functions [10].

In a previous work [11], we have demonstrated that very low dosages of activated solutions of interleukin (IL)-12 and interferon (IFN)-gamma, co-delivered by oral route to experimental asthmatic mice, are able to ameliorate their pathological condition.

In this paper we investigate how oral administration of very low doses of the anti-inflammatory cytokine IL-10 and an antibody raised against the pro-inflammatory cytokine IL-1 alpha confers protection against experimentally-induced inflammation in a mouse model of colitis.

## Materials and Methods

### Animals

All experiments were performed using BALB/c mice weighting 20 - 22 g (10 - 12 weeks of age). Chronic colitis was induced in mice by oral administration of 2% DSS in drinking water for three cycles [12]. Each cycle consisted of 2% DSS dissolved in drinking water for 7 days, followed by a 14 days interval with normal water administration.

For studies on the effect of anti-IL-1 antibody and interleukin-10 on DSS-induced chronic colitis, three groups of 10 mice/group were used. Ten days after completion of the last DSS cycle, one group of mice received 50 fg/kg GUNA interleukin-10 (GUNA s.p.a., Milano, Italy) plus 50 fg/kg GUNA anti-IL-1 antibody (GUNA) (total volume administered: 200 µL) per os, twice a day for 10 consecutive days; the second group of mice received only three cycles of treatment with DSS, as previously described, and no any further treatment, apart from hydro-alcoholic solution vehicle, twice a day for 10 consecutive days; the last group of animals (untreated control group) received normal drinking water only, during the whole time of the experiment, and then the hydro-alcoholic solution vehicle, twice a day for 10 consecutive days (Table 1).

**Table 1:** Treatment Scheme for Induction of the Chronic Colitis Model, in the Different Treatment Groups

Groups Cycles	Group 1: UNTR (10 mice)	Group 2: DSS (10 mice)	Group 3: DSS + IL-10/anti-IL-1 (10 mice)
Cycle 1 dd 1 - 7	Control group: only normal drinking water	DSS 2% in water	DSS 2% in water
Interval dd 8 - 21		Normal drinking water	Normal drinking water
Cycle 2 dd 22 - 28		DSS 2% in water	DSS 2% in water
Interval dd 29 - 42		Normal drinking water	Normal drinking water
Cycle 3 dd 43 - 49		DSS 2% in water	DSS 2% in water
Interval dd 50 - 58		Normal drinking water	Normal drinking water
Treatment dd 59 - 68 (Twice a day)	Hydro-alcoholic solution vehicle	Hydro-alcoholic solution vehicle	50 fg/kg GUNA interleukin-10 plus 50 fg/kg GUNA anti-IL-1 per os

### Evaluation of colonic inflammation

Plasma samples were collected from each mouse before sacrifice, for IL-10 cytokine evaluation. At the end of treatment the intestines of mice were excised and carefully rinsed with saline buffer. The colon was cut close to the ileo-cecal valve and rectum, and the length was measured in 10 mice/group. At necropsy, the macroscopic appearance of the colon (inflammatory score), based on the degree of inflammation and the presence of edema and/or ulcerations, stool consistence, and visible fecal blood, was scored separately on a scale of 0-3 (intestinal bleeding score), (Table 2). Murine colon specimens of 10 mice/group were fixed in 10% neutral buffered formalin, embedded in paraffin, sectioned at 4 µm and collected on xilanized slides. Histopathological analysis using hematoxylin-eosin-stained sections of the proximal and distal colon samples of mice was performed. This histopathological analysis was aimed at evaluating DSS-induced changes in the gut mucosa, such as loss of crypts and reduction of goblet cells, signs of surface epithelial regeneration, focal ulcerations, moderate infiltration of inflammatory cells into the mucosa, and edema in the sub-mucosa: all these parameters were defined by a histological score (scoring 0 - 4), as delineated by Table 3 [12]. Samples were observed with a Nikon Eclipse 80i microscope, equipped with a digital Nikon DS-L1 camera. Another set of murine colon specimens (10 mice/group) were collected for organoculture, 10 mm colon samples were collected in 25 cm^3^ flasks with 3 mL of culture medium (RPMI 1461; 0.1% foetal bovine serum; 1% antibiotics; Euroclone) and incubated for 24 hours at 37 °C in controlled atmosphere (5% CO_2_).

**Table 2:** Scoring System for the Comparative Analysis of Intestinal Bleeding

Intestinal bleeding score	Stool consistency	Blood presence
0	Normal	Negative
1	Soft but still formed	Positive hemoccult
2	Very soft	Blood traces in stool visible
3	Diarrhoea	Rectal bleeding

**Table 3:** Scoring System for Inflammation-Associated Histological Changes in the Colon

Histological score	Histologic changes
0	No evidence of inflammation
1	Low level of inflammation with scattered infiltrating mononuclear cells (1-2 foci)
2	Moderate inflammation with multiple foci
3	High level of inflammation with increased vascular density and marked wall thickening
4	Maximal severity of inflammation with transmural leukocyte infiltration and loss of goblet cells

### Enzyme-linked Immunosorbent assay

We quantified the secretion of several cytokines in murine plasma samples (IL-10) and in organocultures supernatants. We used commercially available enzyme-linked immunosorbent assay kits for quantification of murine IL-12, TNF-alpha, IFN-gamma (all from Pierce Biotechnology, Rockford, IL), KC (R&D Systems, Milano, Italy), IL-17 (BioLegend, San Diego, CA) and IL-10 (Pierce-Euroclone, Pero, Italy).

### Evaluation of intestinal permeability

In order to measure the colon permeability, organ segments (5 mice/group) were mounted between the two chambers of an Ussing System (0.125 cm^2^ opening). Both the mucosal and serosal sides of the chamber were connected to sterilized circulating reservoirs containing 10 ml of oxygenated Krebs buffer (115 mM NaCl, 8 mM KCl, 1.25 mM CaCl_2_, 1.2 mM MgCl_2_, 2 mM KH_2_PO_4_, and 225 mM NaHCO_3_; pH 7.35). The buffers were maintained at 37 °C by a heated water jacket and were circulated by a gas lift column of 95% oxygen/5% CO_2_. The trans-epithelial electrical potential difference (in millivolt) across the mucosal membrane was measured directly, while the trans-membrane resistance was calculated indirectly as ohms × cm^2^, using Ohm’s law.

### Immunofluorescence analysis

We performed immunofluorescence staining for occludin and ZO-1 proteins in colon sections of 10 mice/group. Murine colon specimens were fixed in 10% neutral buffered formalin, embedded in paraffin, sectioned at 4 µm thickness and collected on xilanized slides; samples were deparaffinized, rehydrated, and incubated for 10 minutes at 37 °C in a humidified chamber with protease type XIV 2 mg/mL in TrisHCl buffer; samples were then incubated with glycine 0.1 M for 20 minutes at room temperature, washed with TrisHCl + Triton X-100 0.01%, incubated with NaBH_4_ 0.5 mg/mL for 20 minutes at room temperature, washed with Tris HCl + Triton X-100 0.01%, incubated with Image-IT FX Signal Enhancer (Invitrogen SRL, San Giuliano Milanese, Italy) for 30 minutes, blocked with 2% goat serum (Dako, Glostrup, Denmark) for 20 minutes at room temperature and incubated with rabbit anti-occludin or anti-ZO-1 antibodies (both from Zymed Laboratories, San Francisco, CA) 4 µg/mL. Incubation with secondary antibody Alexa 546 (Invitrogen SRL) goat anti-rabbit and staining of the nuclei with 4’, 6’-diamidino-2-phenylindole (DAPI, Sigma-Aldrich) were then performed. Samples were observed with a Nikon Eclipse 80i microscope equipped with a digital Nikon DS-L1 camera.

In addition, acquired images of ZO-1 and occludin immunofluorescence reactions were used for evaluation of structural integrity of the colon: the percentage of healthy portions of epithelium and crypts, which maintained the expression of tight junction protein, was calculated by the use of the software Image Pro-Plus (version 4.5.019, Media Cybernetics). Evaluation was performed by 3 independent observers.

### Statistical analysis

Student’s t test (paired two-tailed) and GraphPad Prism software (Graph-Pad) were used for comparisons between groups. Values of P less than 0.05 were considered significant.

## Results

### Use of low doses of interleukin-10 plus anti-IL-1 antibody protects against DSS-induced alteration of colonic permeability

**Figure 1:**
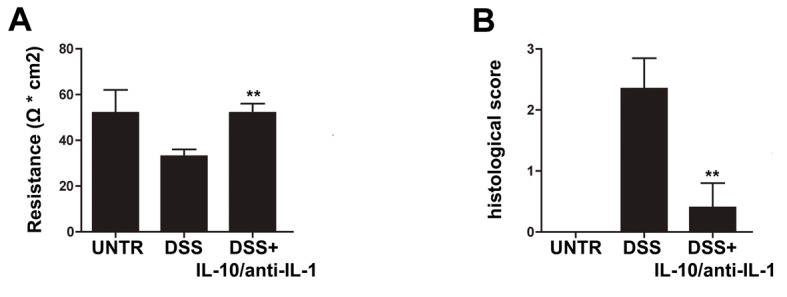
Protective effects of interleukin-10/anti-IL-1 mix in chemical-induced chronic colitis. (A) Evaluation of colon resistance (expressed as Ω * cm^2^) by Ussing chamber analysis in different treatment groups. UNTR (untreated, hydroalcoholic solution only); DSS (DSS 2% plus hydroalcoholic solution); DSS + IL-10/anti-IL-1 (DSS 2% + interleukin-10 IL-10 plus anti-IL-1 antibody). The graph represents mean values ± S.D. (n = 10) Statistical analysis: DSS + IL-10/anti-IL-1 vs. DSS ** P = 0.0028. (B) Evaluation of the histologic score in different treatment groups. UNTR (untreated, hydroalcoholic solution only); DSS (DSS 2% plus hydroalcoholic solution); DSS + IL-10/anti-IL-1 (DSS 2% + interleukin-10 plus anti-IL-1 antibody). The graph represents mean values ± S.D. (n = 10) Statistical analysis: DSS + IL-10/anti-IL-1 vs. DSS ** P = 0.0062.

We evaluated the effects of treatment with low doses of interleukin 10 and anti-IL1 antibody on intestinal permeability in the model of chronic colitis by means of Ussing Chamber apparatus. As shown by Figure 1, DSS-treated mice presented a significant reduction of resistance with respect to control animals. The Ussing chamber data demonstrated a complete protection from the DSS-induced barrier damage in mice receiving interleukin 10 and anti-IL-1 antibody (Fig. 1A).

### Interleukin-10 and anti-IL-1 antibody at low dosage reduce experimental colonic inflammation

**Figure 2:**
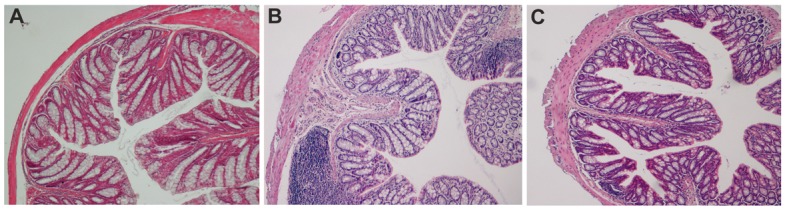
Ematoxilin-Eosin staining of colon sections in chemical-induced chronic colitis. A: histological analysis of paraffin embedded colon sections. UNTR (untreated, hydroalcoholic solution only); B: DSS (DSS 2% plus hydroalcoholic solution); C: DSS + IL-10/anti-IL-1 (DSS 2% + interleukin-10 plus anti-IL-1 antibody).

As shown in Figure 1B and in Figure 2, in the chronic colitis model, treatment with interleukin-10 and anti-IL-1 protected against DSS-induced colonic macroscopical and microscopical inflammation. In particular, the histological score of murine colon samples, which was set at level 0 in control mice, increased to a mean level of 2.35 in DSS-treated animals, and was reduced to 0.4 after mice treatment with interleukin 10 and anti-IL-1 antibody mix solution. Comparable results were also obtained as for intestinal bleeding: in particular, mean score for DSS-treated mice was 2.4 (meaning a very soft stool consistency and the presence of visible blood traces in the faeces), while the mean score of group which received interleukin 10 and anti-IL-1 antibody mix was comparable to the control group, scoring 0 (indicating normal stool consistency with no blood traces).

### Low doses of Interleukin-10 and anti-IL-1 antibody reduce pro-inflammatory cytokine secretion in experimental colitis

**Figure 3:**
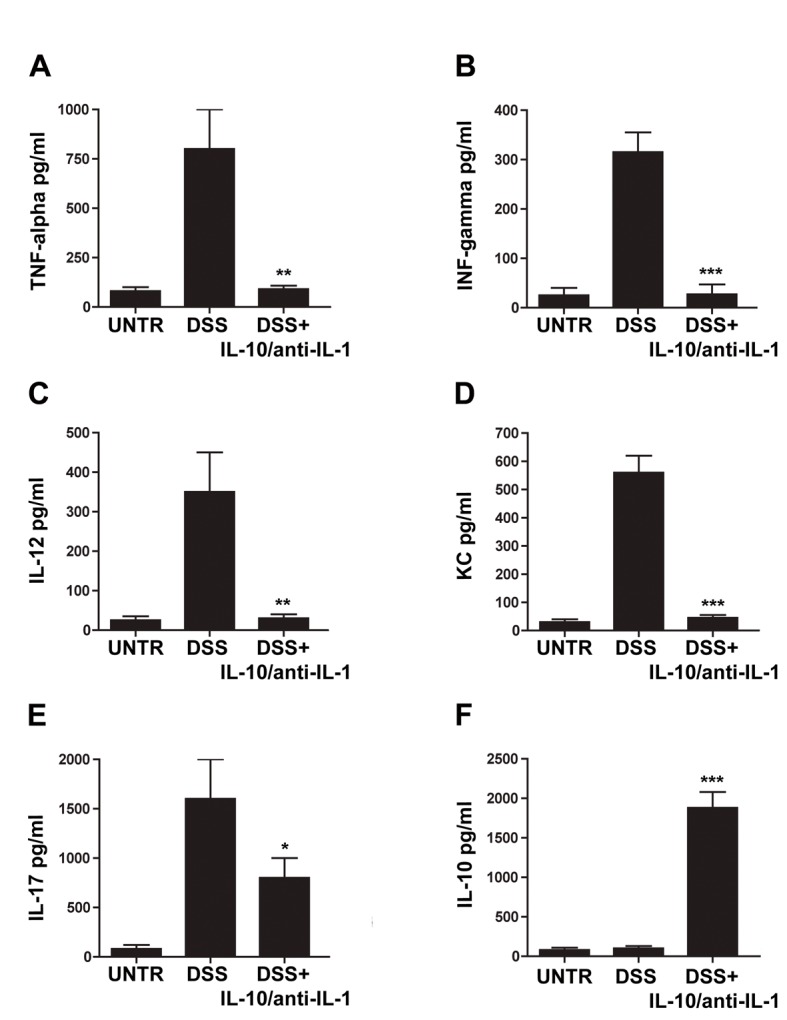
Evaluation of cytokine secretion in chemical-induced chronic colitis. ELISA assays for concentrations of TNF-alpha (A), IFN-gamma (B), IL-12 (C), KC (D), IL-17 (E) and IL-10 (F) in different treatment groups. UNTR (untreated, hydroalcoholic solution only); DSS (DSS 2% plus hydroalcoholic solution); DSS + IL-10/anti-IL-1 (DSS 2% + interleukin-10 plus anti-IL-1 antibody); data are represented as mean values ± S.D. (n = 10). Statistical analysis: A) DSS+ IL-10/anti-IL-1 vs. DSS ** P = 0.0036; B) DSS + IL-10/anti-IL-1 vs. DSS *** P = 0.004; C) DSS+ IL-10/anti-IL-1 vs. DSS ** P = 0.0053; D) DSS + IL-10/anti-IL-1 vs. DSS *** P = 0.0001; E) DSS + IL-10/anti-IL-1 vs. DSS * P = 0.036; F) DSS + IL-10/anti-IL-1 vs. DSS *** P = 0.0001.

To assess whether the treatment resulted in a modulation in the secretion of proinflammatory mediators, organoculture supernatants levels of IL-12, TNF alpha, IFN gamma, KC and IL-17 as pro-inflammatory cytokines and IL-10 as the prototype anti-inflammatory or regulatory cytokine were evaluated: in fact, all these cytokines have been shown to be involved in the DSS colitis inflammatory state [13-15]. The evaluation of the inflammatory cytokine levels in samples of animals treated with interleukin-10 and anti-IL-1 antibody showed a substantial reduction in the concentration of several pro-inflammatory molecules, and, in particular, of IL-12, TNF alpha, IFN gamma, IL-17 and KC (Fig. 3). Accordingly, IL-10 plasma levels are enhanced in treated mice. Plasma level of IL-10 is 7-fold higher than interleukin-10 administered amount, this data allow us to exclude that circulating IL-10 is derived from the administered pool of recombinant cytokine, demonstrating that interleukin 10 and anti-IL-1 antibody mix is able to promote an anti-inflammatory environment (Fig. 3).

### Colonic epithelial barrier structures are protected by interleukin-10 and anti-IL-1 antibody

**Figure 4:**
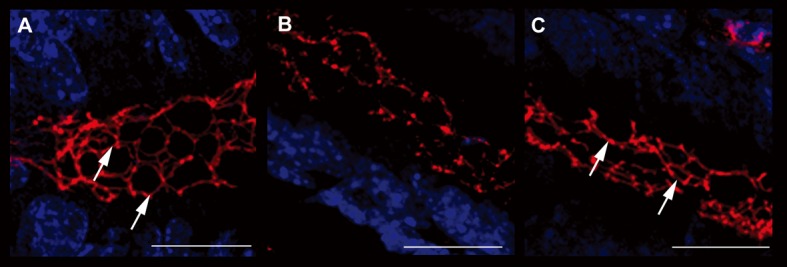
Evaluation of ZO-1 expression. Immunofluorescence anti-ZO-1 (red staining) experiments in the colon samples of different treatment groups showed the different epithelial barrier TJ structural integrity (arrows). A relevant example for each treatment group is shown: A (UNTR; untreated, hydroalcoholic solution only); B (DSS; DSS 2% plus hydroalcoholic solution); C (DSS + IL-10/anti-IL-1; DSS 2% + interleukin-10 plus anti-IL-1 antibody) Bars: 20 µm.

**Figure 5:**
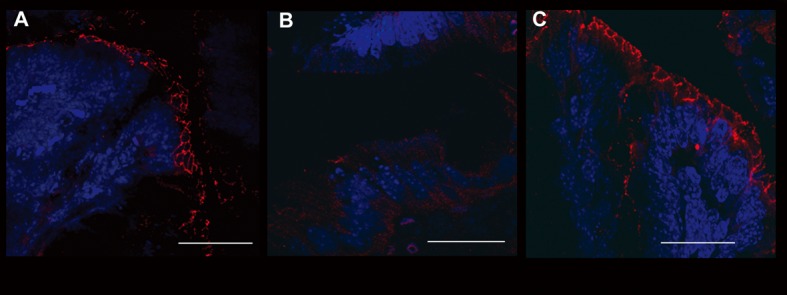
Evaluation of occludin expression. Immunofluorescence anti-occludin (red staining) experiments in the colon samples of different treatment groups showed the different epithelial barrier TJ structural integrity. A relevant example for each treatment group is shown: A (UNTR; untreated, hydroalcoholic solution only); B (DSS; DSS 2% plus hydroalcoholic solution); C (DSS + IL-10/anti-IL-1; DSS 2% + interleukin-10 plus anti-IL-1 antibody). Bars: 20 µm.

In order to confirm the data obtained through the evaluation of colonic permeability, we decided to analyse the expression of occludin and ZO-1, proteins belonging to the junctional complexes [16], in colon tissues of different treatment groups. The immunofluorescence analysis of occludin and ZO-1 localization showed a down- and de-regulation of the junctional morphological structure in DSS-treated mice. Notably, the immunofluorescence analysis of mice treated with interleukin-10 and anti-IL-1 antibody mix revealed that the expression of both TJ proteins along the colon epithelium was maintained, in a similar manner to untreated controls, with a continuous expression in the junctional complexes between epithelial cells along the crypt units, outlining the cell-cell contacts. When crypts were cut transversely, occludin and ZO-1 showed the characteristic continuous honey-comb pattern at the cell membrane. Quantitative evaluation of structural integrity of the colon, in terms of percentage of healthy portions of epithelium and crypts, which express the ZO-1 TJ protein, showed that mice treated with low doses of interleukin 10 and anti-Il-1 antibody were comparable to control untreated mice, in terms of maintenance of an healthy structural state (90.23 ± 10.21% and 79.78 ± 15.22% respectively), while mice of the DSS group where severely damaged at structural level (17.33 ± 8.12% and 21.66 ± 7.65% respectively. DSS vs. DSS+IL-10/anti-IL-1: P = 0.0043). The same quantitative evaluation of structural integrity was performed for occluding expression (UNTR: 92.34 ± 11.46%; DSS: 13.67 ± 8.31%; DSS + IL-10/anti-IL-1: 87.72 ± 7.52%. DSS vs. DSS + IL-10/anti-IL-1: P = 0.0149). These data further sustain the results obtained by Ussing chamber analysis, since they show that the maintenance of the physiological epithelial barrier resistance in mice treated with low doses of interleukin-10 plus anti-IL-1 antibody is linked to a conserved protein expression pattern at TJ level (Fig. 4, 5).

## Discussion

In this work we have shown a new potential therapeutical strategy to treat DSS-induced experimental intestinal inflammation. Our novel approach is based on the administration, by oral route, of compounds able to modulate intestinal immunity at very low dosage by interacting with the mucosal cytokine balance, such as interleukin-10 and an antibody neutralizing IL-1. Oral delivery might offers a means of engaging the cytokine network, to induce beneficial effects in animals and humans [17, 18]; in addition oral administration of cytokines seems to give better results with respect to other routes [19]. The cytokines network in IBD is a complex, dynamic system that plays an important role in regulating mucosal and immune responses [20]; in murine IBD distinctive disease-specific cytokine profiles exist, with significant correlations to disease activity and duration of disease.

Our therapeutical approach is based on a combined interventional strategy: to use a molecule with anti-inflammatory properties, together with an antibody which targets and blocks a pro-inflammatory cytokine. We decided to administer low doses of IL-10 since we aimed to obtain efficacy without inducing side-effects. Given that the pathological condition considered and the molecules used were completely different from the ones used in our previous work on asthma, the transposition of equimolar dosages to this study was a mere starting point, since we did not have any data of literature to support this initial choice, which therefore might result to be wrong.

Interestingly, since we observed an increase in IL-10 plasma levels in treated animals, it could be argued that the administration of low doses of this cytokine could stimulate endogenous IL-10 production by the organism. In this regard, recent works [21-24] have reported that therapeutical approaches based on the stimulation of endogenous immune response of the host are highly effective in promoting the resolution of several inflammatory conditions.

The use of neutralizing anti-IL-1 antibody might have decreased the levels of this cytokine at epithelial barrier level without blocking directly IL-1 epithelial receptor. In fact the use of IL-1 receptor blockers like Anakinra is connected with Crohn’s disease worsening as suggested by Carter et al [25].

Since it is known that IL-1 increases the permeability of tight junctions of intestinal epithelial cells, we have speculated that the blockade of this cytokine might constitute a potentially useful approach to counteract one of the leading etiological events of colitis and Crohn’s disease. Previous experiences of antibody-based therapies for Crohn’s diseased have focused on anti-TNF-alpha antibodies, even if some issues have been raised regarding short- and long-term safety profile of this class of drugs [26, 27]. On the other hand, anti-IL-1 antibodies have proven to be safe for clinical use [28, 29] and effective against DSS-induced acute colitis [30]. This was observed also in our murine models, which did not show side effects following IL-1 antibody administration, both at the lowest and highest tested doses. Our data suggests that IL-1 antibody administration is effective also I chronic models of DSS-induced colitis when co-administered with IL-10.

It has been demonstrated in different works [31-36] that systemic administration is not a safe and efficient way to deliver cytokines and antibodies to specific inductive sites, while their administration via the oral route offers an effective alternative to systemic application, for ease of dispensation. Increasing evidence suggests that oral administration of certain cytokines and antibodies is not only safe and effective, but also avoids the deleterious consequences of systemic administration, retaining sufficient biological activity to affect immunomodulatory functions beyond the local mucosa [10]. As regards the efficacy of orally administered antibodies, which seems to be a very interesting topic at the moment, a recent report by Ochi et al [37] showed that oral was as effective as intravenous administration of anti-CD3 in reversing established experimental autoimmune encephalomyelitis. Therefore, the oral route for cytokine and antibody administration certainly represents one of the more innovative aspects of our proposed novel therapy for colitis.

As the low doses of interleukin-10 and anti-IL-1 antibody demonstrated able to prevent the DSS-induced increase in colonic permeability, in the last part of our work we aimed to evaluate the effect of this therapy on tight junction integrity, by analysing two typical junctional proteins: ZO-1 and occludin. The observed preservation of tight junctions could be linked to anti-IL-1 antibodies. In this regard, a previous work has demonstrated that oral administration of an anti-IL1 antibody was successful in the elimination of IL-1-induced junctional damage [35]. It is of note that the preservation of tight junctions has been observed on 2 different proteins: occludin and ZO-1. Since TJ are constituted by multiple protein complexes, each one of which contributes to the maintenance of the epithelial barrier in a different way, the ability of our therapeutical approach to furnish protective activity on different molecules of these structures seems important. In particular, ZO-1 is involved in signal transduction at cell-cell junctions, while occludin is thought to be involved in the regulation of paracellular permeability and cell adhesion. Future studies will be needed to analyse the eventual protective activity of our therapy on other proteins of TJ complexes, such as claudins and JAM-A.

The protection of epithelial integrity we have obtained is fundamental: in fact, an emerging model of the pathogenesis of Crohn’s disease suggests that 3 essential factors are involved: a breakdown in intestinal barrier function [22]; exposure of luminal contents to immune cells in the lamina propria [38]; and an exaggerated immune response [11]. Patients with CD with clinically active disease have increased intestinal permeability, and in patients with inactive disease, increased intestinal permeability is predictive of clinical relapse. Therefore a breakdown or impairment of the epithelial barrier has been implicated as a critical determinant in the predisposition to intestinal inflammation [39].

Oral administration of low doses of interleukin-10 plus anti-IL-1 antibody mix reduced the general inflammatory status of DSS-treated mice, as shown by reduction of TNF-alpha, IFN-gamma, IL-12 and KC levels. This is fundamental for recovery from IBD condition, given that blockade of proinflammatory mediators is currently considered a promising therapeutic approach for the control of IBD-related mucosal inflammation [9]. Interestingly, we also observed an increase of endogenous IL-10 levels following IL-10 and anti-IL-1 administration. This might be due to the activation of a complex immune defence system, based on the activation of DC that are able to increase the number of Treg cells, which produce IL-10. A direct regulatory IL-10-induced mechanism might be involved, too, as this cytokine has been demonstrated to induce Treg polarization in vivo and in vitro [40, 41].

Our data demonstrate that a mix of interleukin-10 and anti-IL1 antibody at low dosage was successful in preserving colonic permeability, restoring TJ integrity and, at the same moment, in creating an anti-inflammatory environment. The proposed treatment could represent an interesting therapeutic strategy to cure IBD-related intestinal inflammation.
